# Septic Shock Caused by Small Bowel Ischemia in Eosinophilic Granulomatosis With Polyangiitis

**DOI:** 10.1002/ueg2.70093

**Published:** 2025-08-06

**Authors:** Vlad Pavel, Patricia Mester, Florian Weber, Martina Müller, Stephan Schmid

**Affiliations:** ^1^ Department of Internal Medicine I, Gastroenterology, Hepatology, Endocrinology, Rheumatology, and Infectious Diseases University Hospital Regensburg Regensburg Germany; ^2^ Institute of Pathology University of Regensburg Regensburg Germany

**Keywords:** eosinophilic granulomatosis, eosinophils, polyangiitis, shock, small bowel ischemia

We present the case of a 36‐year‐old patient admitted with acute onset diarrhea and abdominal pain. The patient had a known medical history of asthma. Initial laboratory parameters revealed leukocytosis (32.24/nL) with 52% eosinophils, elevated levels of C‐reactive protein (155 mg/L), procalcitonin (3.22 ng/mL) and lactate dehydrogenase (3108 U/L). Due to rapid clinical deterioration and septic shock, the patient was transferred to the intensive care unit. Computed tomography revealed pneumatosis intestinalis in the small bowel (Figure 1a). Although this radiological sign can also indicate a benign disease [1], the clinical condition in our case suggested bowel ischemia. Emergency laparotomy was performed, revealing small bowel ischemia (Figure 1b). Ischemic segments were resected, and a temporary ileostomy was constructed.

**FIGURE 1:**
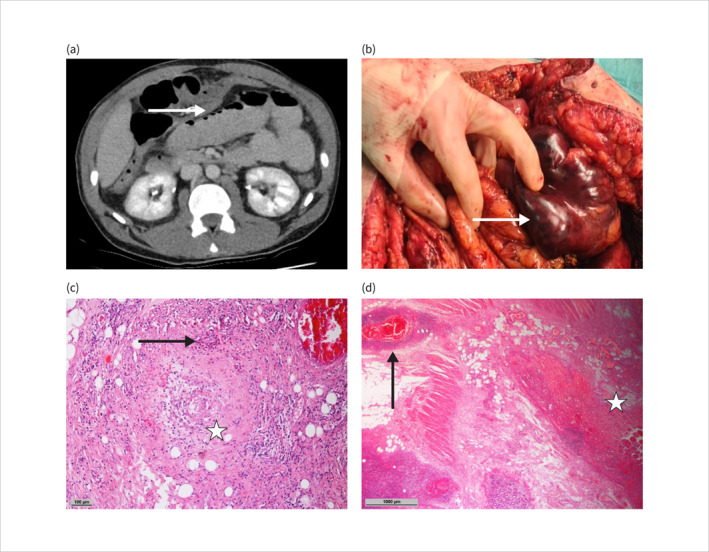
CT showing pneumatosis intestinalis of the small bowel (a). Intraoperative necrotic intestinal loops of the small bowel (b). Histopathology of necrotic bowel segment showing obliterated vessel (asterisk) with eosinophilic infiltrate (arrow) (c, H&E 100x magnification) and acute vasculitis (arrow) with ischemia of adjacent mucosa (asterisk) (d, H&E 20x magnification).

Histopathological examination of the resected bowel revealed vasculitic changes consistent with eosinophilic granulomatosis with polyangiitis (EGPA) (Figure 1c,d): acute vasculitis with ischemia of the corresponding mucosal area and obliterated vessels with an infiltrate of abundant eosinophilic granulocytes. The patient recovered well and, after 1 week in intensive care, was transferred to a general ward where induction therapy with cyclophosphamide and corticosteroids was initiated.

EGPA, formerly known as Churg‐Strauss syndrome, was first described in 1951. It is characterized by disseminated necrotizing vasculitis with extravascular granuloma formation, occurring almost exclusively in patients with asthma and tissue eosinophilia [2]. Clinical manifestations typically include marked peripheral eosinophilia, asthma, chronic sinusitis, cardiomyopathy, pulmonary infiltrates, gastrointestinal symptoms, and peripheral neuropathy [3]. Although gastrointestinal involvement is recognized, bowel infarction is a rare but potentially life‐threatening complication [4]. This diagnosis should be considered in patients with asthma or known EGPA who present with abdominal symptoms.

## Consent

Written informed consent for publication from the patient was obtained.

## Conflicts of Interest

The authors declare no conflicts of interest.

## Data Availability

The data that support the findings of this study are available on request from the corresponding author. The data are not publicly available due to privacy or ethical restrictions.
